# Non-prescribed antibiotic dispensing practices for symptoms of urinary tract infection in community pharmacies and accredited drug dispensing outlets in Tanzania: a simulated clients approach

**DOI:** 10.1186/s12875-022-01905-6

**Published:** 2022-11-19

**Authors:** Pendo M. Ndaki, Martha F. Mushi, Joseph R. Mwanga, Eveline T. Konje, Stella Mugassa, Msilikale W. Manyiri, Stanley M. Mwita, Nyanda E. Ntinginya, Blandina T. Mmbaga, Katherine Keenan, Wilber Sabiiti, Mike Kesby, Fernando Benitez-Paez, Alison Sandeman, Matthew T. G. Holden, Stephen E. Mshana

**Affiliations:** 1 Department of Biostatistics, Epidemiology and Behavioral Sciences, School of Public Health, Catholic University of Health and Allied Sciences, P.O. Box 1464, Mwanza, Tanzania; 2 Department of Microbiology and Immunology, Weill Bugando School of Medicine, Catholic University of Health and Allied Sciences, P.O. Box 1464, Mwanza, Tanzania; 3 School of Pharmacy, Catholic University of Health and Allied Sciences, P.O. Box 1464, Mwanza, Tanzania; 4 National Institute for Medical Research, Mbeya Medical Research Centre, P.O. Box 2410, Mbeya, Tanzania; 5 Kilimanjaro Clinical Research Institute, Kilimanjaro Christian Medical Centre and Kilimanjaro Christian Medical University College, P.O. Box 2236, Moshi, Tanzania; 6 Geography and Sustainable Development, University of St Andrews, North Street, St Andrews, KY16 9AL UK; 7 Division of Infection and Global Health, School of Medicine, University of St Andrews, North Street, St Andrews, KY16 9AL UK

**Keywords:** Antibiotic, Antibiotic resistance, Dispensing practice, Prescription

## Abstract

**Background:**

Antibiotic dispensing without prescription is a major determinant of the emergence of Antimicrobial Resistance (AMR) which has impact on population health and cost of healthcare delivery. This study used simulated clients describing UTI like symptoms to explore compliance with regulation, variations in dispensing practices and drug recommendation, and quality of seller-client interaction on the basis of the gender of the client and the type of drug outlets in three regions in Tanzania.

**Method:**

A total of 672 Accredited Drug Dispensing Outlets (ADDOs) and community pharmacies were visited by mystery clients (MCs). The study was conducted in three regions of Tanzania namely Kilimanjaro (180, 26.79%), Mbeya (169, 25.15%) and Mwanza (323, 48.07%) in March–May 2020. During data collection, information was captured using epicollect5 software before being analyzed using Stata version 13.

**Results:**

Overall, 89.43% (CI: 86.87–91.55%) of drug sellers recommended antibiotics to clients who described UTI like symptoms but held no prescription and 58.93% were willing to sell less than the minimum recommended course. Female clients were more likely than male to be asked if they were taking other medications (27.2% vs 9.8%), or had seen a doctor (27.8% vs 14.7%), and more likely to be advised to consult a doctor (21.6% vs 9.0%); pharmacies addressed these issues more often than ADDOs (17.7% vs 13.2, 23.9% vs 16.6%, 17.7 vs 10.9% respectively). Sellers recommended 32 different drugs to treat the same set of symptoms, only 7 appear in the Tanzanian Standard Treatment Guidelines as recommended for UTI and 30% were 2nd and 3rd line drugs. ADDO sellers recommended 31 drug types (including 2nd and 3rd line) but had permission to stock only 3 (1st line) drugs. The most commonly suggested antibiotics were Azithromycin (35.4%) and ciprofloxacin (20.5%). Azithromycin was suggested more often in pharmacies (40.8%) than in ADDOs (34.4%) and more often to male clients (36.0%) than female (33.1%).

**Conclusion:**

These findings support the need for urgent action to ensure existing regulations are adhered to and to promote the continuing professional development of drug sellers at all outlet levels to ensure compliance with regulation, high quality service and better antibiotic stewardship.

**Supplementary Information:**

The online version contains supplementary material available at 10.1186/s12875-022-01905-6.

## Background

Since 1940, antibiotics (ABs) have been powerful tools in the clinical management of bacterial diseases [1] and while the development of new ABs has slowed, use has increased massively [2]. Between 2000 and 2015, daily global consumption of ABs increased by over 60% from 21.1 to 34.8 billion daily defined doses (DDDs) [3]. In 2015 the annual global ABs consumption was an estimated 42 billion and is projected to rise 200% by 2030, with the majority of the increase being in low and middle income countries [3]. The high consumption of ABs, particularly when without prescription, is a growing public health concern because it is associated with the development and spread of antimicrobial resistance (AMR) [4]. Such resistance (and the slow development of alternative ABs) means that potentially lifesaving drugs become ineffective. It has been previously estimated that if action is not taken to address this issue, by 2050 there may be 10 million AMR related deaths worldwide, costing 100 trillion USD [5]. While this figure could be underestimated, low and middle income countries (LMICs) are expected to face the greatest excess deaths and highest health care cost [6] in large part because of their poor economies, limited health care systems and high rates of infectious diseases [7]. The current extent of AMR in LMICs is difficult to quantify due to a limited number of available studies [8].

Although the social determinants of the development of AMR are complicated and multi-layered, widespread overuse and/or misuse of ABs in human (and animal) health care is among the main contributing causes of AMR [9]. About 80% of global antibiotic consumption occurs outside hospital settings [10], with over 50% of these community-based ABs purchased from community-based drug outlets or informal retailers, often dispensed without prescription [10]. In most nations, ABs are not ‘over the counter’ (OTC) non-prescription drugs, yet in many LMIC settings they are frequently dispensed in response to client demand or seller suggestion, accelerating AB misuse and overuse [11]. Access to Abs without prescriptions is a major factor in the development of AMR, facilitating self-medication with ABs and their improper use e.g., incorrect dosage [12]. Despite adequate levels of regulations in most LMICs there is a lack of enforcement [13], hence dispensing ABs OTC is common among all types of drug sellers [3].

In Tanzania ABs are prescription only drugs, but OTC ABs dispensing is frequent [14]. As part of a broader 3-country interdisciplinary study on the drivers of AMR in East Africa (Holistic Approach to Unravel Antibacterial Resistance in East Africa – HATUA - consortium) [15] we have previously reported high OTC provision of ABs across our 3 study sites in Tanzania (94.6%), with very low numbers of sellers asking clinically relevant questions or offering advice related to ABs stewardship (11.75%) [16]. Using a similar methodology to the one described below, Phase 1 study determined how sellers responded to a direct request for amoxicillin from a client who did not have a prescription and who initially did not describe their symptoms. Also, the study determined if a client’s request for ‘a few days’ worth’ would induce sellers to dispense less than the regulation minimum course; overall, selling without prescription in the Phase 1 study was 93%.

The Phase 1 study demonstrated variation between regions and between urban and rural locations but over 80% of sellers in each location sold amoxicillin OTC without a prescription. It also compared the practices of the two types of drug outlets (type 1 pharmacies or type 2 pharmacies/Accredited Drugs Dispensing Outlets - ADDOs) mandated by the Tanzania Medicine & Medical Devices Authority (TMDA) [17]. The former are intended to operate under the supervision of a registered pharmacist, and can sell all categories of retail medicines: narcotics and psychotropic substances, prescription only medicine, pharmacy only medicines, over the counter (OTC) medicines, and general sales medicines [18]. ADDOs meanwhile, are supervised by any person with medical education background (e.g nurses, clinicians etc) who has attended a 5 weeks’ ADDO training course [19] and so can dispense only a more restricted range of drugs, particularly those that are prescription only (e.g. amoxicillin capsule/suspension, benzyl-penicillin powder for injections, chloramphenicol eye drops, Trimethoprim/Sulfamethoxazole suspension, doxycycline capsules/tablets, Phenoxymethylpenicillin suspension/tablets and procaine penicillin fortified) [20]. The previous/phase I study recorded that fewer pharmacies than ADDOs sold amoxycillin OTC in most regions, but in no region did fewer than 77% of pharmacies do so [16].

The current paper describes Phase 2 of the study. It determines the quality of the seller/client interaction and the range of ABs sold OTC without a prescription to clients presenting with symptoms of UTI (rather than requesting a named AB). It examines whether sellers are prepared to allow clients to determine the length of drug course, by dispensing ABs in quantities below the minimum course recommended in the Tanzania Standard Treatment Guidelines (TSTGs) [20]. Finally, the paper investigates variations in practices on the basis of the gender of the client, and between types of outlets in Mwanza, Mbeya and Kilimanjaro regions.

## Methods

### Study design, setting and duration

A multicenter cross-sectional study which was conducted in community drug outlets between March and May 2022. The study was conducted in three regions of Tanzania namely Mbeya region (Mbeya rural and Mbeya Urban districts) Kilimanjaro region (Moshi rural and Moshi urban district), and Mwanza region (Sengerema rural, Nyamagana and Ilemela districts). The regions were purposively selected to cover South-western, North-eastern, and North-western mainland Tanzania respectively. The main economic activities in Mwanza are subsistence farming, fishing and livestock keeping, while Mbeya and Kilimanjaro is mainly subsistence farming. Nyamagana and Ilemela district form the city of Mwanza which is the second largest city in Tanzania. The study involved community Drug outlets namely community pharmacies and ADDOs which have been re-categorized by Tanzania Medicine & Medical Devices Authority (TMDA)as Part 1 and Part II pharmacies respectively. Community pharmacies are supervised by licensed pharmacist and allowed to sell all types of antibiotics with prescription. On the other hand, ADDOs are supervised by drug dispensers who have undergone a 5 week training on dispensing drugs including antibiotics and allowed to sell limited number of antibiotics with prescription such as amoxicillin capsule/suspension, benzyl-penicillin powder for injections, chloramphenicol eye drops, Trimethoprim/Sulfamethoxazole suspension, doxycycline capsules/tablets,phenoxy methylpenicillin suspension/tablets and procaine penicillin fortified. *(“The Tanzania Food, Drugs and Cosmetics(Schedulingof Medicines)Regulations, 2015”(GN.No.63 of 2015*).

### Study sampling procedures and sample size

Sampling procedure started with mapping all the community pharmacies and ADDOs that were located in the three regions of study. Mapping used Global Positioning System (GPS) conducted by trained fieldworkers who systematically went across roads pedestrian area taking coordinated and recoding location, type of outlets and observed regulatory compliance [21].

Visited drug outlets in the current study drug outlets were selected randomly from the near 100% sampling frame generated in phase 1 of the HATUA project in 2019 (621 in Mwanza, 304 in Mbeya and 232 in Kilimanjaro) [22]. This study therefore randomly involved about 50% of the mapped community drug outlets i.e. a total of 672 different sellers in Kilimanjaro (180, 26.79%), Mbeya (169, 25.15%), and Mwanza (323, 48.07%).

### Data collection

The current study applied a mystery client study, sometimes referred to as a simulated client study, to assess dispensing of practices Research assistants (MCs) entered in the community pharmacies and drug shops as sick clients in need of pharmacy services prior to presenting with UTI symptoms. MC method reduces response bias hence investigate practice in real life. Furthermore, observations through mystery client survey is the only rigorous and valid method to document actual behaviour-in-context (in this case, dispensing behaviour/practices of the drug sellers) which cannot be documented by other methods such as questionnaire survey which gives ‘reported’ rather than ‘actual’ behaviour/practices [3]. Thought the participants were not consented at the exact time of data collection, but there was a prior information through fliers that drug outlets will be visited for research purpose through using MC method within 3 months.

The full scenario used by the mystery clients (MCs), shown in supplementary material 1, tested the dispensing practices of sellers presented with a client without a prescription, who described symptoms of urinary tract infection. Using everyday language, the MCs presented with low grade fever, painful micturition, urgency and pyuria and only if questioned, reported the symptoms had been coming and going for the past 1 month and had not responded to amoxicillin treatment. If offered amoxicillin, the MCs asked ‘for something stronger’ since amoxicillin ‘had not worked’. Only if further questioned, MCs reported that they had only taken a few days’ worth of amoxicillin. MCs accepted whatever recommendation and treatments the seller advised although if a drug was recommended, they made one request to purchase ‘only a few days’ worth’, before then accepting whatever course the seller recommended. If the seller agreed to this request, we recorded that the MC had received a ‘half course’. In pharmacotherapeutic terms, exactly what constitutes a full course of antibiotic would depend on the drug dispensed and is stipulated in the TSTGs [20]. Having established what drug and course a seller recommended, the MC either bought or declined to purchase, depending on the price.

After withdrawing and before moving to the next premises, MCs used digital tablets or mobile phones and the software Epicollect 5 [21] to record their findings. MCs recorded the questions asked and advice given by the seller, the medications recommended and in cases where drugs were purchased, the price and quantity of tablets sold. In addition, they recorded the GPS of the premises, and made an attempt to observe or subjectively assess compliance with some of the key minimum guidelines mandated by Tanzanian Guidance on drug sales (i.e.: minimum age of seller should be 18 or over, they should wear uniform and ID badge, display evidence of qualification/certification to practice, and the premises should be in good order and good repair [20].

### Quality control procedures

The research tool was initially developed by the PI and Co-investigators in English language. Then it was used in in the training of research assistants (Final year bachelor of pharmacy students) who contributed their inputs. . Then, the developed English version was translated into Kiswahili by Co-investigators in collaboration with the research assistant before being pretested. After the pretesting of the tool and accommodating reformulations arising from pretest, it was back-translated into English and the final English and its Kiswahili version were obtained for data collection in the main study. Development of tools and pretesting was also considered as part of the training of the research assistants.

Eleven MCs (3 females and 8 males) were trained on the study, its objectives, ethics, and protocols and on the mystery client scenario. To ensure a consistent simulation and data standardization MCs rehearsed the scenario and practiced common behavior and responses with lead researchers familiar with clinical and pharmacy settings. Through the surveying period, lead researchers reviewed the data daily, addressing any inconsistencies with the MC concerned, and met with all MCs twice a week for feedback and updating.

### Data management and analysis

Collected data were downloaded from Epicollect5 and analyzed in Stata version 13 (Stata Corp., College Station, TX, USA) [23]. After data cleaning and consistency checks, the results were analyzed using descriptive univariate and bivariate statistics. The data were summarized and results compared across regions, by type of outlet (ADDOs and community pharmacies) and by clients’ gender. Chi square or fishers exact was used to measure the strength of association observed between outlet types and clients’ gender. We calculated an additive score for each seller based on whether they asked key questions or gave key advice relevant to good AB stewardship. The questions contributing to this score were: “Did the seller…”

1. …probe you and get you to describe the second set of symptoms (e.g., that you had cloudy, smelly, and bloody pee)?
2. … ask if you had experienced these symptoms before?
3. …ask if you were taking any other medication?
4. …ask if you had seen a doctor?
5. …ask if you had a prescription?
6. …suggest you did not need any drug treatment?
7. …recommend that you see a doctor and/or get a prescription?
8. …at any point mention or advise you about the issue of antibiotic resistance?

Each question was weighted equally and carried 1 mark if the MC marked ‘yes’, 0 if the MC marked ‘no’. This resulted in an index ranging from 0 to 8 which was used in some analyses.

## Results

A total of 672 drug outlets were visited by MCs during the study (113 pharmacies and 559 ADDOs). Estimated compliance with mandated regulation was high or very high for some operational attributes; less than 2% of all those serving the MCs were estimated to be younger than 18 years old (see Table 1). Meanwhile, MCs assessed that 75% of premises in all regions were in ‘adequate’ condition (on a 3-point scale), with Kilimanjaro having the least in ‘poor’ and the most in ‘excellent’ condition. The majority of sellers displayed certificates of qualifications (74% in Kilimanjaro, 64% in Mbeya and 59% in Mwanza). However, the wearing of ID badges was a rare practice (less than 2%) across all three regions (Table 1).

**Table 1:** Profile of drug sellers and drug outlets visited by region

Characteristics		Kilimanjaro *n* = 180 n (%)	Mbeya *n* = 169 n (%)	Mwanza *n* = 323 n (%)	Overall *n* = 672 n (%)
Gender	**Female**	157 (87.22)	141 (83.43)	229 (70.90)	527 (78.42)
	**Male**	23 (12.78)	28 (16.57)	94 (29.10)	145 (21.58)
Age	**< 18 yrs**	5 (2.78)	1 (0.59)	1 (0.31)	7 (1.04)
	**> = 18 yrs**	175 (97.22)	168 (99.41)	322 (99.69)	665 (98.96)
Drug outlet level	**ADDO**	161 (89.44)	137 (81.07)	261 (80.80)	559 (83.13)
	**pharmacy**	19 (10.56)	32 (18.93)	62 (19.20)	113 (16.82)
Organization	**Poor**	1 (0.56)	15 (8.88)	31 (9.60)	47 (6.99)
	**Adequate**	134 (74.44)	136 (80.47)	237 (73.37)	507 (75.45)
	**Excellent**	45 (25.00)	18 (10.65)	55 (17.03)	118 (17.56)
Display certificates	**No**	47 (26.11)	64 (37.87)	131 (40.56)	242 (36.01)
	**Yes**	133 (73.89)	105 (62.15)	192 (59.44)	430 (63.99)
Seller wore ID	**No**	176 (97.78)	168 (99.41)	315 (97.52)	659 (98.07)
	**Yes**	4 (2.22)	1 (0.59)	8 (2.43)	13 (1.93)

*ADDO* Accredited Drug Dispensing Outlet, *ID* Identification (such as a name tag)

### Quality of seller’s interaction with client by outlet level and client’s gender

The quality of interaction between client and seller, measured by questions asked or advice offered by seller, was generally low with 89.7% of sellers scoring between 1 and 3 on our 8-point scale and only 10.3% scoring between 4 and 7 (Table 2). Proportionally, sellers in pharmacies were more likely than those in ADDOs to address any one of the items on the behavior or advice checklist. Critically however, only in a minority of cases were clients asked one of the three most important questions relevant to dispensing ABs for the treatment of UTI or to give the most important advice, i.e.: ‘have you seen a doctor’ (17.9%), ‘do you have a prescription’ (6.1%) and ‘you should see a doctor to get a prescription’ (12.1%). Again, those in pharmacies were proportionately more likely to address these items than those in ADDOs (23.9 vs 16.6%, 10.6 vs 5.1 and 17.7% vs 10.9% respectively). However, while pharmacies performed better as a sector than did ADDOs, over three quarters of pharmacies failed to ask these important questions. Proportionally, female clients were more likely to be asked if they were taking other medications (27.2% vs 9.8%) or had seen a doctor (27.8% vs 14.7%) and were more often advised to consult a doctor (21.6% vs 9.0%).

**Table 2:** Antibiotic dispensing practices and quality of seller’s interaction with client by outlet level and client’s gender

Action of seller	ADDOs (total ***n*** = 559)	pharmacies (total ***n*** = 113)	female clients (total ***n*** = 162)	male clients (total ***n*** = 510)	Clients overall (total ***n*** = 672)
	n (%)	n (%)	n (%)	n (%)	n (%)
**Quality of interaction**					
1. Asked about past experience of symptoms	227 (40.61)	57 (50.44)	73 (45.06)	211 (41.37)	284 (42.26)
2. Asked about taking other medication	74 (13.24)	20 (17.70)	44 (27.16)	50 (9.80)	94 (13.99)
3. Asked if client had seen a doctor	93 (16.64)	27 (23.89)	45 (27.78)	75 (14.71)	120 (17.86)
4. Asked about prescription	29 (5.19)	12 (10.62)	9 (5.56)	32 (6.27)	41 (6.10)
5. Suggested no need for a drug	10 (1.79)	7 (6.19)	1 (0.62)	16 (3.14)	17 (2.53)
6. Suggested client see a doctor/get prescription	61 (10.91)	20 (17.70)	35 (21.6)	46 (9.02)	81 (12.05)
7. Mentioned AMR	41 (7.33)	22 (19.47)	6 (3.70)	57 (11.18)	63 (9.38)
**Unwilling to dispense**					
• Total who did not recommend a pharmaceutical drug/were unwilling to sell	56 (10.02)	15 (13.27)	21 (12.96)	50 (9.80)	71 (10.57)
**Willing to dispense**					
• Willing to sell but only full-course	156 (27.91)	49 (43.36)	43 (26.54)	162 (31.76)	205 (30.51)
• Willing to sell half-course but advice full-course	176 (31.48)	21 (18.58)	55 (33.95)	142 (27.84)	197 (29.32)
• Willing to sell half-course without any question	171 (30.59)	28 (24.78)	43 (26.54)	156 (30.59)	199 (29.61)
**Total willing to sell a pharmaceutical drug**	503 (89.98)	98 (86.73)	141 (87.04)	460 (90.20)	601 (89.43)

The results on dispensing practice record that the majority of sellers 89.4% (CI: 86.9–91.5%) displayed a willingness to dispense antimicrobials OTC to clients who described UTI like symptoms and held no prescription. No significant difference (*p* = 0.304) was observed between ADDOs (90.0%) and pharmacies (86.7%) or between clients’ gender, in the propensity to dispense without prescription. In response to the MCs request for only ‘a few days’ worth’ of drugs, two-thirds of sellers were willing to sell a ‘half course’ to the client, either with (29.3%) or without providing further advice (29.6%). Proportionally more pharmacies than ADDOs insisted that MCs bought a full course (43.4% v 27.9%) see Table 2.

### Patterns of antibiotics dispensed

A total of 601 sellers recommended 33 different antimicrobials to treat the same set of UTI-like symptoms described by MCs. The most commonly suggested antimicrobials were Azithromycin (213/601, 35.4%) and ciprofloxacin (123/601, 20.5%) which belong to the macrolide and quinolone class respectively and both are in the ‘watch’ category. Only 7 of the drugs recommended by sellers were listed in the TSTGs as suitable to treat the UTI -like symptoms presented by MCs. In ADDOs, Ciprofloxacin (2nd line treatment) was the most commonly proposed drug (110/503, 21.9%) followed by Cephalexin (1st line treatment) (56/503, 1.1%). While in pharmacies cephalexin was the most commonly recommended drug (15/98, 15.3%) followed by ciprofloxacin (13/98, 13.3%).

Of the suggested drugs which are not recommended for UTI treatment by TSTG, Azithromycin was proposed by 34.4% (173/503) of ADDOs and 40.8% (40/98)of pharmacies. Doxycycline was suggested more by ADDOs (45/503, 9.0%) than pharmacies (2/98, 2.0%). Only 10% (3/33) of the suggested antibiotics were allowed to be stocked by ADDOs. Across the wide variety of drugs that sellers suggested, we analyzed whether the sex of the MC predetermine the type. a combination of ‘ciprofloxacin and doxycycline’ was significantly suggested to more male (24/480,5.0%) than female clients (1/121, 0.83%) (*p*-value of 0.040). Slightly, more women (57 /121,47.1%) than men (201/480, 41.9%) were recommended for drug listed in the TSTGs as suitable for the treatment of UTIs p-value 0.302. While, azithromycin, was recommended slightly to more male clients (173/480, 36.1%) than female (40/121, 33.1%) *p*-vlue 0.269.

## Discussion

The score of quality of interaction between sellers and clients with 10.3% of drug sellers presenting between 4 and 7 questions or advice to clients. Majority of sellers (89.4%) were willing to sell ABs without prescription for clients with UTI symptoms. There was no significant difference between community pharmacies and ADDO, and between male and female clients as far as quality of interaction in concerned. Azithromycin(35.4%) and Ciprofloxacin(20.4%) were the commonest dispensed antibiotics.

As part of a wider, two phase, three-country investigation of the drivers of AMR in East Africa, this study has documented the role played by private drug shops in the ABs provision landscape in three sites in Tanzania (Mwanza, Mbeya and Kilimanjaro regions). Tanzania’s pharmacy (prescription handling and control) regulations prohibit the dispensing of ABs without prescription [19]. The study has presented the how different kinds of drug sellers, in different contexts, would respond to a client who did not have a prescription, reporting symptoms of UTI and only if questioned, a history of similar symptoms and self-medication.

The results of this survey suggest that compliance with certain areas of drug provider regulation is relatively good; most premises displayed certificates of qualification and were in at least adequate condition to store and sell medications, and only a small minority may have been staffed by personnel below the minimum required age. Furthermore, the study records relatively good client/seller interaction scores compared to phase 1 of our study (in which MCs made a straightforward request for a specific drug – amoxicillin, without necessarily describing UTI-like symptoms). In that phase interaction seemed poor and transactional, with only 10.6% addressing 1–3 clinically relevant questions or treatment suggestions on our 8-point scale and only 1.2% addressing between 4 and 7 [16]. By comparison in phase 2, reported here, when presented with symptoms and a request for advice, 90% of sellers addressed between 1 and 3 clinically relevant questions/advice items and 10% scored between 4 and 7. Similarly, on the critically important question ‘do you have a prescription’, scores were better in response to scenario 2/phase 2 but only marginally so (6.1% compared to 1.2% in scenario 1).

However, notwithstanding the improved ‘quality of interaction’ in response to a client’s request for advice rather than demand for a named drug, these scores were dominated by questions related to patients’ illness history: ‘have you experienced these symptoms before’ (42.3%), and to a lesser extent: ‘are you taking other medication’ (14.0%). Meanwhile, propensity to ask the questions and give the advice most relevant to both regulation and AMR stewardship remained worryingly low (‘do you have a prescription’ 6.1%, ‘have you seen a doctor’ 17.9%, ‘you should see a doctor/get a prescription’ 12.1%, ‘you should take or finish a whole course’ 9.4%). This is particularly noteworthy given that all sellers are officially mandated to sell antibiotics only if a prescription is provided. While the pharmacy sector performed better than ADDO level sellers, two thirds of pharmacies still failed to address these most clinically/AMR relevant questions in our 8-point checklist. Concern that improved interaction does not necessarily indicate improved antibiotic stewardship seem to be confirmed by the fact that most sellers (89.7% CI: 87.2–91.8% with little difference between the sectors), dispensed an antimicrobial OTC despite clients not being in possession of a prescription (only slightly fewer than the 94.6% who dispensed amoxicillin in response to a direct request in phase 1 of the study [16]).

Our findings corroborate those of another study conducted in the Moshi urban district of Kilimanjaro region which found that 92% of pharmacies dispensed antimicrobials to clients without prescription [24], but do so for a larger sample in a larger number of areas of Tanzania. Furthermore, this study went beyond assessing if sellers would dispense without prescription and in addition investigated whether sellers would sell less than the minimum course mandated by the TSTGs. We found that 58.9% of sellers (with little difference between pharmacy and ADDO sectors) were willing to dispense ‘just a few days’ worth’ of a drug. This is less than the 93.1% who sold a ‘half course’ in phase 1 but remains a dangerously high figure given the possible association between under dosing and the development of AMR.

In line with a previous study in Moshi urban [24], the current study also found that azithromycin was the drug most commonly suggested for UTI even though it is not recommended for UTI in the TSGT. Proportionately, more pharmacies than ADDOs inappropriately suggested azithromycin as a UTI treatment (40, 40.8% vs 173, 34.4%) but far more ADDOs than pharmacies suggested doxycycline inappropriately (45,8.9% vs 2, 2.0%). This drug is also not recommended for the treatment of UTI but it is one which ADDOs are mandated to sell.

Antibiotic drugs are classified into three ‘lines’ according to their effectiveness. Second- and third-line drugs are more effective than first-line, but also more toxic with more potential side effects. They should be held in reserve for cases where first-line drugs have failed to clear an infection and/or the pathogen has become resistant to a first- or second-line drug [25]. If the seller asked about illness history, our scenario included a report that the MC had taken amoxicillin, but it ‘had not worked’. At one level it is unsurprising then that only 9 (1.8%) ADDOs sellers and no pharmacists suggested amoxicillin. However, it should be remembered that only 284 (42,3%) asked about illness history and only 94 (14%) about other medication. From this perspective the failure to suggest amoxicillin is noteworthy, as is the fact that only 101 (16.8%) recommended a first line drug for UTI (which included amoxicillin [9, 1.5%], cephalexin [71, 11.81%], trimethoprim/Sulfamethoxazole [14, 2.3%] and nitrofurantoin [7, 1.16]).

Some 134 sellers (35.44% of all types) recommended a second line drug (which included ciprofloxacin [123, 20.5%] and levofloxacin [11, 1.8%]), with a very small number recommending the third-line drug amoxiclav (23, 3.83%). Meanwhile, 290 (49%) sellers suggested antimicrobials that the TSTGs classify as inappropriate for the treatment of UTI. The dispensing of drugs inappropriate to symptoms described suggests that sellers *either* lack familiarity with the TSTGs, *or* neglect them, *or* customize them (e.g., ADDOs selling a drug which is *not* suitable to treat the symptoms described by a client but which they *are* mandated to dispense). It also reinforces that empirical diagnosis of symptoms is a specialist skill which explains why antibiotics should not be dispensed without a doctor’s prescription.

While our scenario together with sellers’ relatively high propensity to ask about ‘illness history’ and ‘other medication’ might explain some of the failure to dispense Amoxicillin, they do not explain why sellers did not recommend the alternative 1st line treatment they are empowered to dispense. Furthermore, even though our methodology meant that if offered amoxicillin, our MCs requested ‘something stronger’, because ‘amoxicillin had not worked’, a thorough questioning of the client would have revealed that they had previously taken ‘only a few days’ worth’ of the drug. Results from phase 1 of our study suggests that sellers *know* that clients habitually take a ‘half course’ because sellers habitually dispense less than the recommended minimum courses. Therefore, not only did ADDO sellers not have permission to sell second-line drugs, but *both* they *and* the pharmacy sellers might reasonably have advised MCs to buy and finish a full course of an appropriate first-line drug before second-line drugs were even considered.

### Strengths and limitations of the study

This study has several methodological strengths: Firstly, the mystery client method allows for more objective assessment of dispensing practice than would self-reported responses to a questionnaire. Secondly, the scenario used allowed us to assess all of the following simultaneously: compliance with general standards, quality of seller/client interactions, range of drugs dispensed in response to a common set of symptoms, and whether sellers would sell antibiotics without a prescription and do so in quantities less than the recommended course. It also allowed us to compare results to an earlier scenario which was based on a simple request for a commonly dispensed drug. Thirdly, the study had a larger sample size and covered more regions than earlier Tanzanian studies.

Limitations of the study were: While more extensive than previous studies in this region, the sample size in this study was only a subset of the 100% sample in our own phase 1 study of the same regions, and like it, is limited in scope to 3 regions of Tanzania. Secondly, our quantitative methods demonstrate patterns of behaviour but do not necessarily give us the drivers for poor practices, which calls for a qualitative approach. Thirdly, the drug outlets were mostly located in urban settings and the sample is dominated by findings from Mwanza.

## Conclusion

In Kilimanjaro, Mbeya and Mwanza regions, Tanzania, this study tested pharmacy and ADDO drug seller responses to mystery clients who presented with symptoms of UTI and asked for advice and revealed only if questioned that they had no prescription and had previously self-medicated. Its findings have implications for the treatment of UTI, the promotion of antibiotic stewardship and for regulatory policy. The study identified, first: a widespread willingness to dispense antibiotics without prescription, and in quantities below the recommended minimum course: Second, that less than half the drugs recommended were listed in the Tanzanian Standard Treatment Guidelines (TSTGs) as suitable for the treatment of UTI: Third, that ADDOs frequently stocked antimicrobials they were not permitted to sell, including 2nd and 3rd line/watch list drugs, and that sellers of all types too readily bypassed the 1st line/access treatment for UTI, amoxicillin, without rigorously questioning whether clients had previously used it or used it appropriately: Fourth, that while pharmacies questioned clients more effectively than ADDOs the sector still underperformed, especially in relation to questions relevant to antibiotic stewardship: Fifth, that while women were both more thoroughly questioned and more likely to have a relevant drug recommended, like men, the majority were offered drugs not recommended for treating UTI in the TSTGs.

Tanzania was the first African country WHO recognized for achieving a functioning regulatory system for medical products [14], yet this study demonstrates a lack of compliance with some of the most important elements of regulation. Recurrent inspection is required to enforce existing regulations on authorized medicines and prescriptions. However, this should be accompanied by continuing professional development that reinforces the importance of WHO AWaRE categories and national standard treatment guidelines on appropriate drugs and minimum course, and which encourages sellers to question and advise clients more thoroughly. Community pharmacies and ADDO sellers need to be encouraged to recognize their role in ensuring the health of their customers and communities. Further qualitative study is required to identify sellers’ perceptions of their current dispensing practices and explore what might motivate their compliance with regulation and improve their stewardship of life saving antimicrobials.

## Supplementary Information

**Additional file 1.**

## Data Availability

The dataset generated and analyzed during the current study is available from correspond author on reasonable request.

## Abbreviations

ABs: Antibiotics
ADDO: Accredited Drug Dispensing Outlets
AMR: Antimicrobial Resistance
DDDs: Daily Defined Doses
GPS: Global Positioning System
HATUA: Holistic Approach to Unravel Antibacterial Resistance in East Africa
LMICs: Low and Middle Income Countries
MCs: Mystery Clients
OTC: Over the Counter
TMDA: Tanzania Medicine & Medical Devices Authority
TSTG: Tanzania Standard Treatment Guideline
UTI: Urinary Tract Infection
