# Supplementary material for: Non-prescribed antibiotic dispensing practices for symptoms of urinary tract infection in community pharmacies and accredited drug dispensing outlets in Tanzania: a simulated clients approach
Source: BMC Prim Care. 2022 Nov 19;23:287. doi: 10.1186/s12875-022-01905-6 (PMC9675131; doi:10.1186/s12875-022-01905-6)
Supplement: Supplementary file 1 — Additional file 1. [file 12875_2022_1905_MOESM1_ESM.docx]

**Supplementary information 1**

**The Mystery Client Scenario.**

**Scenario protocol: The ‘patient’ presented urinary tract infection symptoms to drug sellers without mentioned the required antibiotics.**

1. Tell the sellers that you are not feeling well by presenting symptoms of low grade fever, painful micturition, urgency and pyuria.
2. Only if asked for any other symptoms, tell that your micturition is a bit cloudy, and smells odd, and sometimes blood in the urine.
3. If you are asked questions about your illness (and you may not be) – answer using the appropriate response below – you may need to adapt them a little – but keep the key facts the same:
4. Yes – I have had these symptoms before – about one month ago but they went away then came back
5. Yes – I have been taking something for these symptoms – Amoxicillin which I have bought myself from another drug sellers
6. No – I previously did not take a full course of Amoxicillin – I only took a few days’ worth
7. No – I am not taking any other drugs (apart for some Amoxicillin I previously took for this problem – but that finished 4-5 days ago)
8. No – I do not have a prescription
9. No – I have not been to see a doctor for this (because I was too embarrassed and feared it was an STI).
10. No – I am not pregnant
11. No – I do not wish to have any kind of test today to see what the problem might be – I do not have the time/money/I don’t feel able (whatever works that allows the client not to be tested)
12. If you are offered Amoxicillin say – I don’t want that because it doesn’t seem to work for me anymore – the symptoms keep coming back - can I have something stronger?
13. If you are offered something other than Amoxicillin say – Is that strong? I need something strong.
14. Attempt to by a half dose/course:

For any drug you agree to buy – attempt to buy just a couple of days’ worth – say you want to see if it works.

1. Take the seller’s advice:

After the conversation (see above) you should eventually take the sellers advice i.e. one or more of the following:

- 1. Accept you do not need antibiotics if that is what is advised
  2. Buy whatever is eventually recommended
  3. Buy a half course of the recommended drug if the seller will allow that
  4. Buy a full course of drugs the recommended if the seller says they will not buy a half course.
